# 

**Published:** 1895-04

**Authors:** 


### CONTENTS FOR APRIL, 1895.

ORIGINAL COMMUNICATIONS. PAGE.

Hot Springs, South Dakota ; A Health Resort. By J. W. GROSVENOR, M. D. ... 513
The Lesser Omentum. By BYRON ROBINSON ... 520
Our Insane Patients and Their Hospital Relations. By CHARLES A. RING, M. D ... 525
Atrophy of the Optic Nerve in Diseases of the Nervous System. By ALVIN A. HUBBELL, M. D ... 531

CLINICAL REPORT.

Vesical Calculi in the Female—Report of a Case. By FREDERICK J. MANN, M. D ... 536

REPORTS OF SOCIETIES.

The Eighth Periodical International Ophthalmological Congress ... 537
University of the State of New York ... 541

SELECTION.

The Gold Preparations in Some Skin Diseases and Syphilis ... 546

EDITORIAL.

Pelvic Inflammatory Disease—What is True Conservatism in its Treatment? ... 552
Topics of the Month ... 554

Personal ... 563
Obituary ... 564
Society Meetings ... 564
Book Reviews ... 566
Miscellany and Literary Notes ... 575

